# Enduring musician advantage among former musicians in prosodic pitch perception

**DOI:** 10.1038/s41598-023-29733-3

**Published:** 2023-02-14

**Authors:** Xin Ru Toh, Shen Hui Tan, Galston Wong, Fun Lau, Francis C. K. Wong

**Affiliations:** 1 Linguistics and Multilingual Studies, School of Humanities, Nanyang Technological University, Singapore, Singapore; 2 School of Brain and Behavioral Sciences, The University of Texas at Dallas, Dallas, TX USA

**Keywords:** Human behaviour, Auditory system

## Abstract

Musical training has been associated with various cognitive benefits, one of which is enhanced speech perception. However, most findings have been based on musicians taking part in ongoing music lessons and practice. This study thus sought to determine whether the musician advantage in pitch perception in the language domain extends to individuals who have ceased musical training and practice. To this end, adult active musicians (*n* = 22), former musicians (*n* = 27), and non-musicians (*n* = 47) were presented with sentences spoken in a native language, English, and a foreign language, French. The final words of the sentences were either prosodically congruous (spoken at normal pitch height), weakly incongruous (pitch was increased by 25%), or strongly incongruous (pitch was increased by 110%). Results of the pitch discrimination task revealed that although active musicians outperformed former musicians, former musicians outperformed non-musicians in the weakly incongruous condition. The findings suggest that the musician advantage in pitch perception in speech is retained to some extent even after musical training and practice is discontinued.

## Introduction

Musical training has been associated with various cognitive enhancements^1^, making it an attractive enrichment and intervention activity. In the language domain, one notable finding is that musical training is linked to an advantage in speech perception. For instance, musicians are better than non-musicians at perceiving speech in noisy conditions even in older adulthood^2,3^. In particular, a vast amount of literature has documented positive music-to-language cross-domain transfer effects in pitch processing, which undergirds Patel’s OPERA hypothesis^4^. The OPERA hypothesis describes how musical training benefits the neural encoding of speech when five requirements are satisfied: there is an *overlap* in the brain networks employed to process an acoustic feature common to music and speech; the processing of the shared acoustic feature occurs at higher *precision* in music than in speech; and the musical activities evoke strong positive *emotion*, have frequent *repetition*, and encompass focused *attention*. The OPERA hypothesis may account for the superior pitch processing abilities in speech seen in musically trained individuals, as pitch is a basic acoustic property found in both music and speech. While pitch differences are used to form melodies in music, they are used to convey contrastive meaning via lexical tones, stress, and intonation in speech. To augment the OPERA hypothesis, this study seeks to explicate whether the musician advantage persists in former musicians who have ceased musical training and practice.

The OPERA hypothesis is well supported by empirical studies comparing musicians and non-musicians. Studies have found that among individuals with no tone language experience, musicians outperform non-musicians in lexical tone perception^5–15^. In addition, musicians without tone language experience show enhanced brainstem and cortical encoding when listening to lexical tones^14,16,17^. Yet, it is uncertain whether the musician advantage in lexical tone perception also exists among tone language speakers. It was previously found that for English or French speakers, musicians outperform non-musicians in Cantonese tone discrimination, whereas for Cantonese speakers, musicians and non-musicians both show ceiling effects^18^. On the other hand, it was also found that Cantonese musicians outperform Cantonese non-musicians in the discrimination and identification of merging Cantonese tone pairs, especially the most difficult Tone 2/Tone 5 contrast^19^. More recently, Toh et al.^20^ found that even among speakers of a tone language, those who have received musical training outperform non-musicians in non-native lexical tone perception. Apart from lexical tone perception, studies have found that among individuals with no tone language experience, musicians are better than non-musicians at perceiving stress, which is indicated by a combination of pitch, duration, and intensity variations^21,22^. However, it remains unclear whether the musician advantage in stress perception also applies to tone language speakers. Among English speakers, musicians outperform non-musicians in English stress perception, while Cantonese-English bilingual musicians and non-musicians perform equally well^23^. Tone language experience has been linked to enhanced pitch perception abilities in speech^6,24–30^. As such, one possible explanation for the conflicting findings is that the musician advantage for pitch perception in speech applies across speakers irrespective of language background, but the more subtle effect among tone language speakers is likely to be masked by ceiling-like performance in tasks that are not sufficiently sensitive.

Besides lexical tones and stress, another aspect in which there is mounting evidence for the musician advantage is prosody. A series of studies have consistently found that musicians outperform non-musicians in detecting pitch contour expectancy violations^31–35^. This research paradigm, first created by Schön et al.^31^, is designed by manipulating the fundamental frequency of either the final notes of musical phrases or final words of linguistic phrases. In particular, the weakly incongruous condition entails a small pitch change which is difficult to detect, and hence evaluates pitch perception in a more fine-grained manner. Through both behavioural and electrophysiological measures, they found that adult musicians detected these pitch variations better than non-musicians in not only music but also their native language, thereby lending support for a domain-general pitch processing mechanism. This finding was reinforced in follow-up cross-sectional and longitudinal studies^32,33^, in which they found similar group differences among 8-year-old children, despite the fact that the children musicians received a shorter duration of musical training than the adult musicians in the original study. Their finding was also expanded in follow-up studies introducing unfamiliar language contexts^34,35^, in which they found that participants across groups found it more difficult to detect pitch changes in a non-native language or pseudolanguage than in their native language. The researchers posited that understanding the semantic content and being familiar with intonational contours in sentences might help with anticipating and detecting pitch changes in one’s native language. That said, the researchers found that musicians held an advantage over non-musicians in detecting prosodic pitch violations across native and non-native language contexts. Moreover, behavioural studies have found that musicians outperform non-musicians in matching spoken utterances to their intonation melodies^36^ and identifying emotional prosody in speech^37–39^. Interestingly, similar results were seen in a longitudinal study with 6-year-old children, with those who were randomly assigned to receive 1 year of musical training in the form of keyboard or vocal lessons outperforming those who received no lessons when tested on the identification of emotional prosody in speech^37^. Collectively, these studies substantiate the notion that musical training facilitates speech perception at not only the segmental but also supra-segmental level.

Furthermore, neurological studies suggest that musical training is linked to structural and functional differences in the brain^40–50^. Notably, the effects of musical training on brain development seem to be causal in nature^33,45–47^. For instance, Hyde et al.^46,47^ randomly assigned 6-year-old children without any behavioural or brain differences in pre-tests to receive either 15 months of musical training or no training. They found that only those who received musical training showed structural brain changes in motor and auditory areas which were correlated with behavioural improvements on melodic and rhythmic discrimination tests. These studies suggest that there may be musical training-induced brain plasticity effects that could potentially translate to long-lasting cognitive impacts. While the data on ageing and musicianship remains scant, there is emerging evidence that an age-related decline in auditory perception may be mitigated by musical training among lifelong musicians who maintain regular musical practice. Older and younger adult musicians outperform non-musicians in various auditory processing abilities, such as detecting speech-in-noise and mistuned harmonics, assessed using neurophysiological and behavioural measures^51–53^.

In light of the above findings, musical training does appear to facilitate speech perception, providing empirical evidence for the OPERA hypothesis^4^. A critical question to consider is whether the OPERA hypothesis can be extended to former musicians. Studies on the effects of musical training typically characterise musicians as individuals with ≥ 6 years of musical training and ongoing instrumental practice for ≥ 1 h a week^54^. However, such professional musicians may not represent the general population in which many individuals who take up music lessons in childhood eventually do not commit to it^55,56^. Although there has been extensive research on professional active musicians, more research needs to be done with individuals who choose not to pursue musicianship professionally but nonetheless have had some musical experience. Of particular interest is whether cognitive benefits such as in speech perception persist even after musical training and practice is discontinued. Qualifying the extent of the influence of musical training among individuals who have undergone music attrition will serve to not only provide insight on the generalisability of the OPERA hypothesis, but also inform the effectiveness of musical training as a means of improving cognitive and linguistic abilities in the long-term, as well as protecting against age-related cognitive decline.

As noted by Costa-Giomi^56^, few studies to date have investigated whether cognitive advantages exist in the long term after musical training and practice is discontinued. Costa-Giomi and Ryan^58^ (as cited in Costa-Giomi^57^) conducted a longitudinal study in which children in the experimental group received 3 years of piano lessons. Seven years after musical training was discontinued, the researchers found no differences in IQ or memory between the adults who had and had not received childhood musical training, suggesting that musical training does not result in permanent cognitive benefits. Nevertheless, the researchers postulated that the lack of long-lasting cognitive improvements may have been due to low attendance and time spent practising the musical instrument^55,58^. In contrast, two behavioural studies found improved performance in various cognitive tasks such as IQ^59^ and executive functions^60^ in adulthood even after musical training and practice had ceased, suggesting that musical training has long-term benefits and contributes to the establishment of a cognitive reserve. However, the measures used in these behavioural studies have focused on general cognitive abilities rather than speech perception abilities specifically.

In terms of auditory perception, two brain imaging studies have found that musical training in early childhood provide sustained enhanced neural processing of auditory stimuli in adulthood after musical training and practice had ceased. Skoe and Kraus^61^ found that young adults who had received musical training in childhood showed more robust signal-to-noise ratio brainstem responses to pure tones, as compared to non-musicians. White-Schwoch et al.^62^ found that older adults with a greater number of years of musical training in childhood or young adulthood showed faster neural timing in response to consonant–vowel transitions in speech syllables presented in quiet and noise, compared to older adults with fewer number of years of musical training or no musical training at all. Although these two brain imaging studies suggest that music-related neuroplasticity is maintained even after music attrition, studies have yet to investigate if these neural traces translate to a clear behavioural advantage in acoustic processing of speech stimuli. This is a critical research gap that the present study aims to bridge.

In sum, although the literature has generally established that professional musicians have an advantage over non-musicians in pitch perception abilities in the language domain, it remains inconclusive whether this musician advantage would also be observed behaviourally among individuals who have ceased musical training and practice. That being the case, the overarching aim of our study is to add to the OPERA hypothesis and elucidate whether a potential music-to-language transfer effect exists among former musicians. To this end, our study compared active musicians, former musicians, and non-musicians in their ability to perform a well-replicated experimental task—detecting linguistic prosodic pitch violations.

Although there has been a burgeoning number of studies revolving around various types of pitch perception in speech, such as lexical tones and stress, we were theoretically motivated to focus on prosody for several reasons. Firstly, prosody is often described as “the music of speech”^63^, thereby making it an obvious candidate for the present study on music-to-language transfer. Patel himself has called attention to the fact that both melody in music and prosody in speech rely primarily on the same acoustic parameter of pitch contour, with the former necessitating more precise acoustic processing than the latter^64^. This overlap in neural resources has been demonstrated in the studies outlined above, in which musicians tend to surpass non-musicians in prosodic pitch perception. On top of that, Patel and other researchers have shown that individuals on the other end of the spectrum with a musical disorder known as amusia exhibit deficits in perceiving speech prosody^64–67^. Accordingly, speech prosody is of exceptional relevance to the OPERA hypothesis. Secondly, unlike lexical tones which are only of pertinence to tone languages, speech prosody is an important aspect of all languages, thereby making it a universal topic of interest with great practical significance. Broadly speaking, prosody signals speaker intention and meaning, imparting crucial information pertaining to syntax and pragmatics^68^. Research on first language acquisition has found that prosodic sensitivity is related to literacy skills^69^, reading comprehension^70,71^, and speech comprehension^72–74^. In a similar vein, research on second language acquisition in children and adults has found that prosodic sensitivity might facilitate the learning of word order and new vocabulary^75,76^, while exposure to prosodic features of the target language apparently improves second language proficiency and fluency^77,78^. The findings yielded from this study will therefore have important pedagogical implications for language and literacy skills as well as foreign language learning.

In order to study prosodic pitch perception, we chose to adopt the well-replicated prosodic pitch contour expectancy violation task, as it has consistently demonstrated the musician advantage in different age groups and languages with robust findings. Given that previous studies revealed a trend in which participants, regardless of musicianship, showed superior performance in detecting prosodic pitch violations in a familiar language relative to an unfamiliar language^34,35^, two different language contexts were implemented in the present study. We included a non-native language context in part to help circumvent a problem we anticipated; namely, that tasks using native language stimuli might not be adequately sensitive to tease apart group differences^18,23^, especially for tone language speakers. Furthermore, by introducing both native and non-native language contexts, we hoped to examine music-to-language transfer effects both with and without the top-down influence from other types of linguistic processing, allowing us to better assess the generalisability of the effects. Finally, the two language contexts mirror first language competence and second language learning respectively, shedding light on the practical application of the enduring music-to-language transfer effects in former musicians, if any.

## Method

### Participants

Participants were recruited to take part in the study via an online screening questionnaire. They were between 19 and 42 years old (*M* = 23.04, *SD* = 3.90), with normal hearing based on an audiometric test (25 dB HL for octave frequencies from 500 to 4000 Hz). All of the participants were either native Singaporeans or had lived in Singapore for at least 10 years to ensure that they were familiar with the local accented variety of English. They had no formal exposure to the French language, the non-native speech stimuli used in this study.

A total of 127 individuals participated in this study. Data from 31 participants was excluded due to the following cases: (a) participants with self-reported exposure to French (*n* = 8); (b) participants who had between 2 and 6 years of musical training experience (*n* = 23).

The final dataset consisted of 96 participants. They were classified into three groups based on information obtained from a self-report questionnaire on their language and music background. In this study, active musicians consisted of those who had had at least 6 years of musical training and were still currently maintaining a consistent practice schedule of at least 3 h per week in the past 2 years (*n* = 22). On the other hand, former musicians referred to those who similarly had at least 6 years of musical training but had stopped maintaining a regular practice schedule for at least 2 years (*n* = 27). Finally, non-musicians referred to those who had had less than 2 years of musical training (*n* = 47). Those with musical training predominantly had experience in string, wind, and vocal musical training. None of the participants were musicians by profession. Reflecting the diversity of multilingualism in the local population, the majority of the participants were proficient in English and Mandarin Chinese (*n* = 86), while several were proficient in English and a second language other than Mandarin Chinese, specifically Malay (*n* = 3), Tamil (*n* = 5), Tagalog (*n* = 1), and Burmese (*n* = 1). The representation of non-Mandarin Chinese speakers was similar across groups, χ^2^(2) = 2.355, *p* = 0.308.

To validate the grouping, participants’ general musical abilities were assessed using the Musical Ear Test (MET)^79^. The MET consisted of two components: the melody subtest and the rhythm subtest. For each subtest, participants listened to 52 pairs of phrases, and had to judge whether the second phrase was the “same” or “different” compared to the first phrase. Half of the trials were “same” trials and the other half were “different” trials. The “different” trials involved a pitch violation in the melody subtest and a rhythmic change in the rhythm subtest. The MET stimuli were delivered via headphones, and participants gave their responses on an accompanying answer sheet. All participants completed the melody subtest followed by the rhythm subtest. Table 1 shows the final sample and descriptive information of each participant group.

**Table 1:** Participant group demographics.

	Participant group			Group differences on musical abilities (MET scores) as revealed by one-way ANOVA
	Active musicians (AM)	Former musicians (FM)	Non-musicians (NM)	
N	22	27	47	N/A
Age	21.45 (2.87)	23.07 (3.93)	23.77 (4.13)	N/A
No. of years of musical training	11.00 (3.80)	9.37 (3.12)	0.09 (0.28)	*F*(2,93) = 202.564, *p* < 0.001 AM > NM (*p* < 0.001) AM = FM (*p* = 0.069) FM > NM (*p* < 0.001)
No. of practice hours per week within the past two years	5.91 (2.51)	0.70 (0.77)	N/A	N/A
No. of years since musical training was discontinued	0.09 (0.29)	5.26 (3.44)	N/A	N/A
MET melody (%)	85.58 (7.43)	77.99 (7.92)	69.23 (7.38)	*F*(2,93) = 37.299, *p* < 0.001 AM > NM (*p* < 0.001) AM > FM (*p* = 0.002) FM > NM (*p* < 0.001)
MET rhythm (%)	78.76 (7.93)	73.29 (7.77)	66.98 (9.49)	*F*(2,93) = 14.579, *p* < 0.001 AM > NM (*p* < 0.001) AM = FM (*p* = 0.093) FM > NM (*p* = 0.010)
Language background	20 English-Mandarin bilingual 1 English-Malay bilingual 1 English-Tagalog bilingual	26 English-Mandarin bilingual 1 English-Tamil bilingual	40 English-Mandarin bilingual 2 English-Malay bilingual 4 English-Tamil bilingual 1 English-Burmese bilingual	

Mean values and standard deviations (in parentheses) are given for age, musical background, and musical abilities. For the group differences on musical abilities, one-way ANOVA and pairwise comparisons are Bonferroni corrected.

## Materials and procedures

The research procedures were approved by the Institutional Review Board at the Nanyang Technological University. All research methods were performed in accordance with the relevant guidelines and regulations. Written informed consent was obtained from all participants and/or their legal guardians before participation.

After providing their written informed consent, participants were seated comfortably in a soundproof booth, and undertook two experimental tasks. Firstly, the participants completed a two-choice speech pitch discrimination task. The English and French language blocks were counterbalanced across participants, with half of the participants presented with the English set first, and the other half with the French set first. Secondly, the participants completed a general musical abilities test, i.e., the MET. Short breaks were given between tasks to prevent fatigue. The total length of time for participation was approximately 1 h, and the participants were monetarily compensated for their time upon successful completion of the experiment.

Participants’ linguistic perception abilities were assessed using a pitch violation discrimination task that has been well-replicated in the literature^31–35^. For the pitch discrimination task, 40 spoken declarative sentences in English and French respectively were recorded to form the experimental speech stimuli (see Supplementary Tables 1 and 2). The sentences were compiled and modified from a combination of sources, including the Harvard Sentences database^80^ for the English stimuli and Smith’s paper^81^ for the French stimuli, with the final word in each sentence being disyllabic as in Marques et al.’s study^34^. Two female speakers, one native in Singapore English and the other in French, voiced the English and French sentences respectively at a normal speaking rate. The recorded sentences were then digitised (sampling at 44.1 kHz and 16 bit) using Audacity^®^ Version 2.0.5.0^82^.

**Figure 1:**
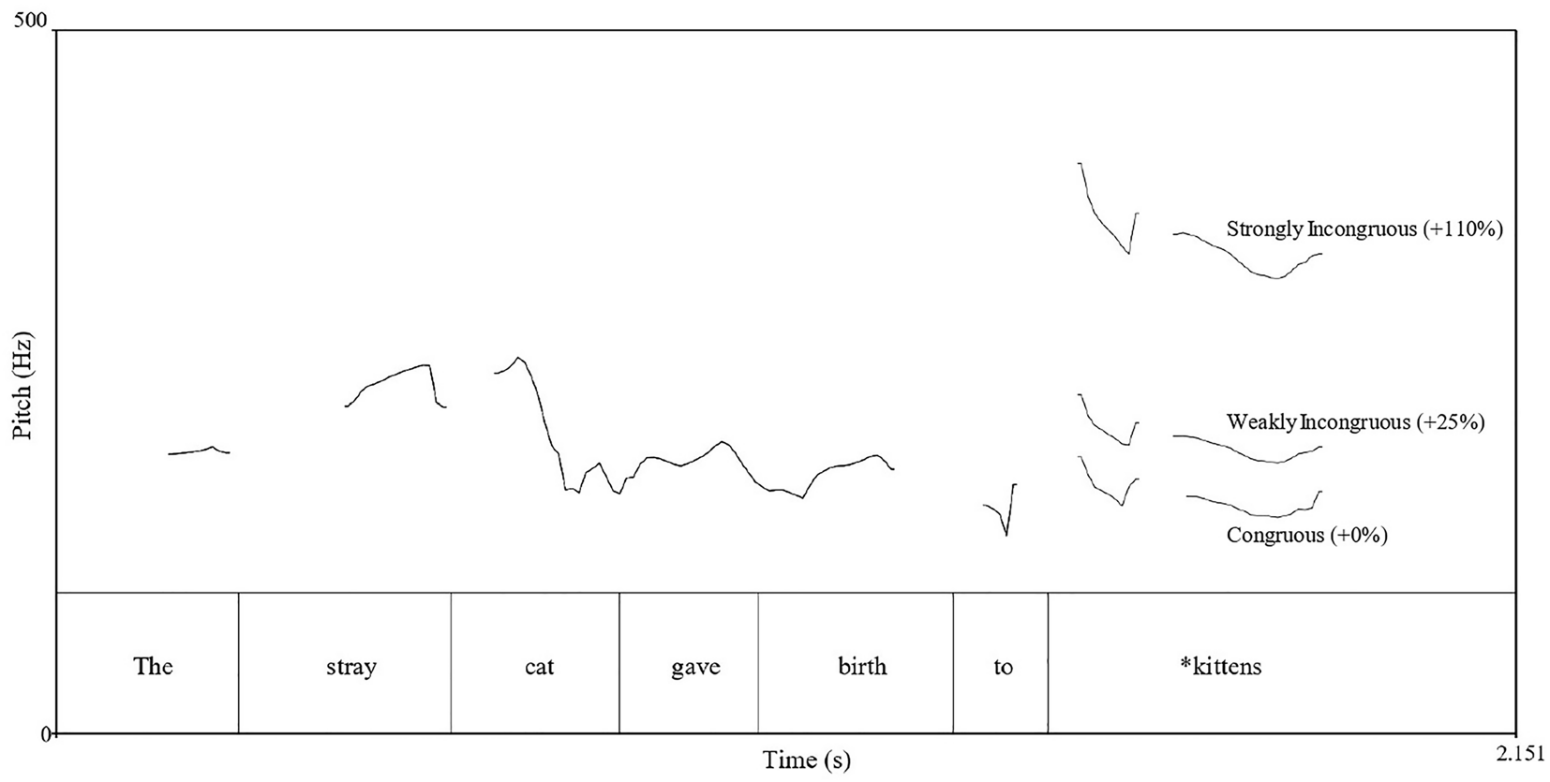
Fundamental frequency (F0 in Hz) for a sample sentence in the three prosodic conditions.

For each language, there were three different auditory conditions, and 40 sentences were presented in each auditory condition, thus leading to a total of 120 sentences. The final word of each sentence was either prosodically congruous, weakly incongruous, or strongly incongruous. In the prosodically incongruous conditions, the pitch (F0) of the final words was increased using Praat^83^, such that there was a local pitch manipulation on the final words (+ 25% in the weakly incongruous condition, + 110% in the strongly incongruous condition) while maintaining the original natural global pitch contour (Fig. 1). The pitch increases used in the present study differ from those used in past studies (+ 35% in the weakly incongruous condition, + 120% in the strongly incongruous condition)^31–35^. Preliminary pilot testing using conventional pitch increase values revealed a ceiling effect among our Singaporean participants, likely because enhanced pitch perception abilities in speech have been associated with bilingualism^84^ and tone language experience^6,24–30^. As such, we reduced the pitch incongruity in order to increase the difficulty of the task, and preliminary pilot testing using our modified pitch increase values obtained pitch discrimination accuracy rates across the experimental conditions which were similar to those found by Marques et al.^34^.

Participants listened to the speech stimuli via headphones. They were briefed that they would be listening to either English or French sentences, and that comprehension of the sentences was not required. In each trial, participants were asked to judge whether the final word of each sentence sounded normal (congruous condition) or strange (weakly incongruous or strongly incongruous conditions). Responses were recorded via a keyboard press, “N” or “S” respectively. Participants were asked to provide a response within 3 s. The practice phase consisted of 6 trials, with feedback provided at the end of each trial to indicate if the participants had answered correctly. The experimental phase consisted of 120 trials, broken up into four blocks of 30 sentences each. Sentence blocks were counterbalanced across participants; half of the participants in each group heard blocks one and two first, while the other half heard blocks three and four first. Sentences from each experimental condition occurred equally frequently within each block and in pseudorandom order. Up to three consecutive “strange” trials were allowed within each block, while pitch-manipulated variants of the same sentence were not allowed to occur within the same block.

## Results

A 2 $\times$ 3 $\times$ 3 mixed ANOVA was conducted with pitch discrimination accuracy as the dependent variable, language (native vs. non-native) and prosodic congruity (congruous vs. weakly incongruous vs. strongly incongruous) as the within-subject factors, and music group (active musicians vs. former musicians vs. non-musicians) as the between-subject factor. As Mauchly’s Test indicated that the assumption of sphericity had been violated for the prosodic congruity effect, χ^2^(2) = 242.497, *p* < 0.001, and the language by prosodic congruity effect, χ^2^(2) = 153.767, *p* < 0.001, Greenhouse–Geisser correction was applied, ε = 0.519 and ε = 0.552 respectively. As Box's M Test indicated that the assumption of equality of covariance had been violated, Box’s M = 249.251, *F* = 5.307, *p* < 0.001, Pillai’s Trace was used. For all pairwise comparisons, Bonferroni correction was applied.

**Figure 2:**
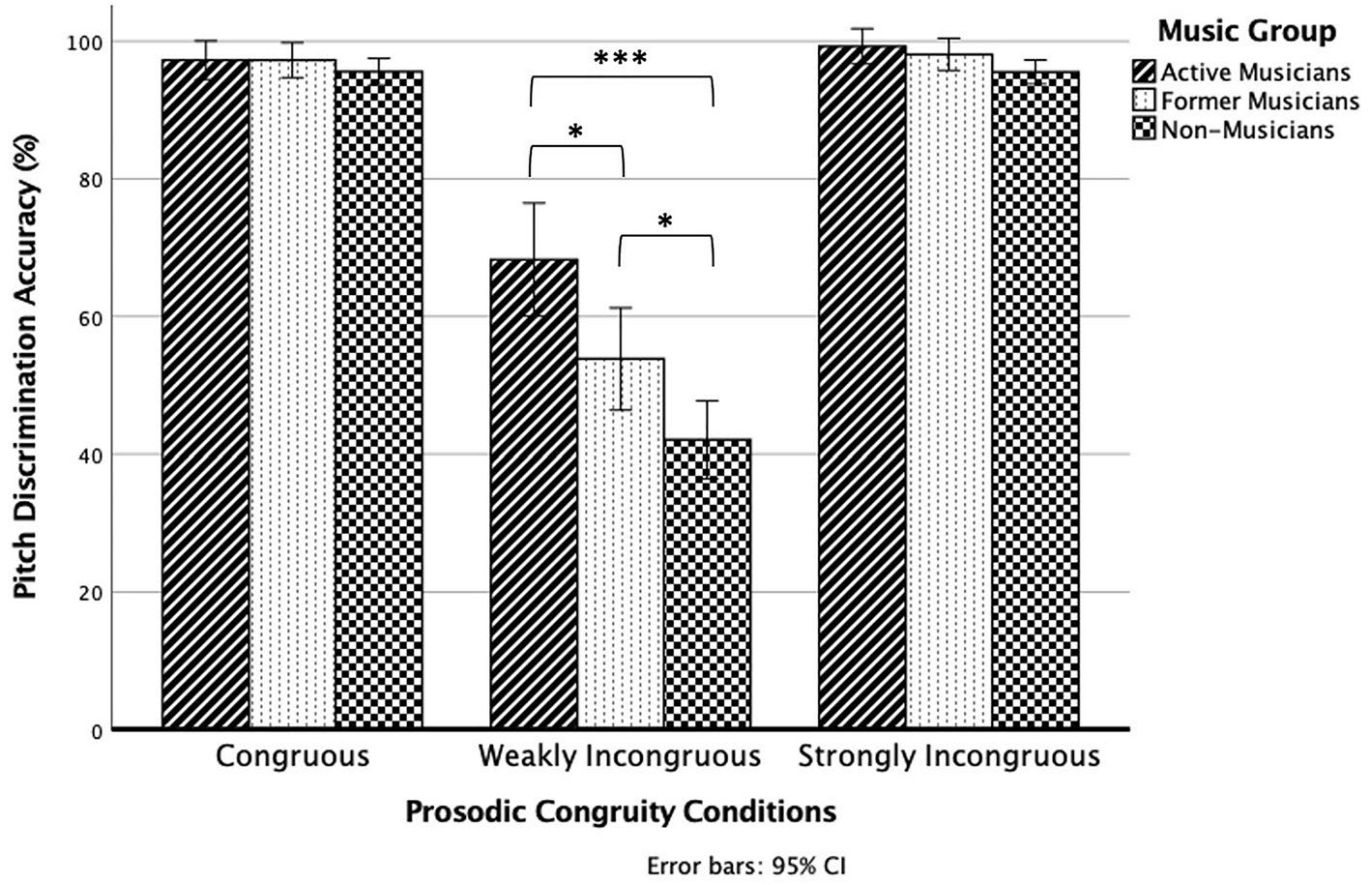
Pitch discrimination accuracy of active musicians, former musicians, and non-musicians in the three prosodic conditions. Error bars denote standard error. *p < 0.05, ***p < 0.001.

The interaction effect between prosodic congruity and music group was statistically significant, *F*(2.074,96.456) = 10.124, *p* < 0.001 (Fig. 2). The main source of the interaction effect as revealed by simple effect analyses was from the weakly incongruous condition, *F*(2,93) = 13.877, *p* < 0.001; the mean pitch discrimination accuracy was significantly different between active musicians (68%), former musicians (54%), and non-musicians (42%). Pairwise comparisons revealed that active musicians outperformed former musicians (*p* = 0.034) and non-musicians (*p* < 0.001), while former musicians also outperformed non-musicians (*p* = 0.042).

The effect of music group was significant for the strongly incongruous condition, *F*(2,93) = 3.293, *p* = 0.042; the mean pitch discrimination accuracy was significantly different between active musicians (99%), former musicians (98%), and non-musicians (96%). However, pairwise comparisons revealed no significant differences between groups after Bonferroni correction. Active musicians did not differ from non-musicians (*p* = 0.060), and former musicians differed from neither active musicians (*p* = 1.000) nor non-musicians (*p* = 0.257). Meanwhile. the effect of music group was not significant for the congruous condition, *F*(2,93) = 0.767, *p* = 0.467.

The three-way interaction between language and prosodic congruity and music group was not significant, *F*(2.207,102.648) = 0.335, *p* = 0.737; neither was the interaction between language and music group, *F*(2.000,93.000) = 0.615, *p* = 0.543.

**Figure 3:**
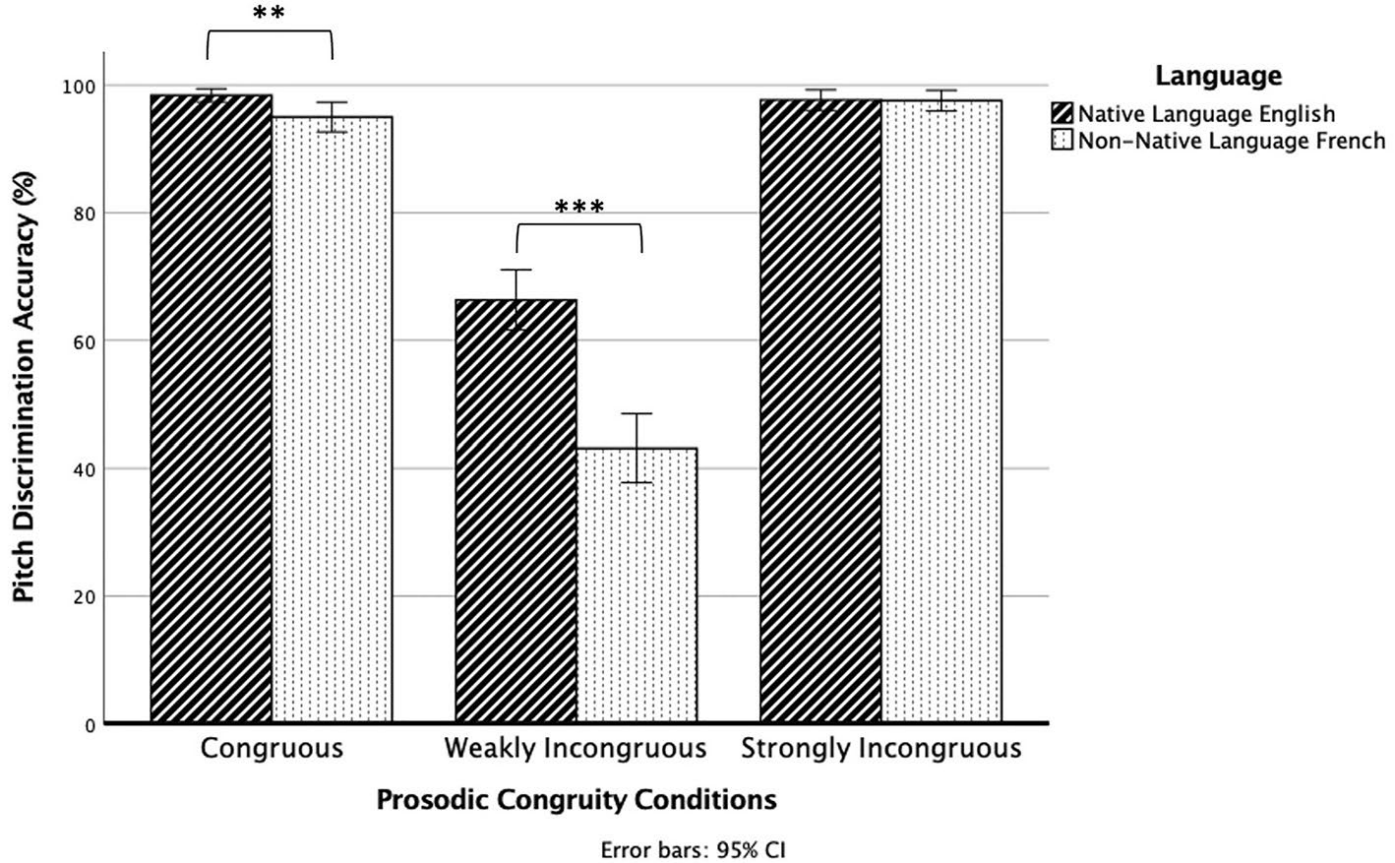
Pitch discrimination accuracy for the native language English and non-native language French in the three prosodic conditions. Error bars denote standard error. **p < 0.01, ***p < 0.001.

There was also a significant interaction effect between language and prosodic congruity, *F*(1.104,102.648) = 39.450, *p* < 0.001 (Fig. 3). The effect of language was significant for the weakly incongruous condition, *F*(1.000,93.000) = 63.833, *p* < 0.001, where participants showed higher pitch discrimination accuracy in their native language English (66%) than in their non-native language French (43%). The effect of language was also significant for the congruous condition, *F*(1.000,93.000) = 8.816, *p* = 0.004, where participants showed higher pitch discrimination accuracy in their native language English (98%) than in their non-native language French (95%). However, the effect of language was not significant for the strongly incongruous condition, *F*(1.000,93.000) = 0.008, *p* = 0.931).

Significant main effects were found for language, *F*(1.000,93.000) = 85.129, *p* < 0.001, prosodic congruity, *F*(1.037,96.456 = 376.582, *p* < 0.001, and music group, *F*(2,93) = 14.275, *p* < 0.001. Participants showed higher pitch discrimination accuracy in their native language English (87%) than in their non-native language French (79%). The weakly incongruous condition (52%) was the most difficult to detect compared to the congruous condition (96%) and strongly incongruous condition (97%). Active musicians (88%) and former musicians (83%) showed higher pitch discrimination accuracy compared to non-musicians (78%).

## Discussion

The present study is one of the first to ascertain whether individuals who have discontinued musical training and practice retain a behavioural advantage over non-musicians in pitch perception abilities in speech. Our key finding is that there was a significant interaction effect between prosodic congruity and music group. In the weakly incongruous condition where pitch deviations were small and difficult to detect, our results showed a stepwise progression in pitch discrimination accuracy, with active musicians having better performance than former musicians, who in turn had better performance than non-musicians.

Our finding of an advantage by musicians over non-musicians in pitch discrimination echoes past findings that musical training facilitates pitch perception in the language domain^5–23,31–39^, thereby pointing towards a common domain-general pitch processing mechanism in music and speech perception as described in the OPERA hypothesis^4^. Our finding also coheres with findings of long-lasting neural changes from past musical training in young adults^61^ and older adults^62^, as well as findings of improved cognitive performance in adulthood even after ceasing musical training^59,60^. Taken together, it appears that former musicians share similar neural enhancement as active musicians, and that the sharpened neural processing translates to perceptual benefits behaviourally. One explanation is that musical training requires individuals to attend to subtle sound contrasts, such as in pitch and duration. Consequently, musicians become more sensitive to such subtle acoustic cues, which has a positive spillover effect when discriminating similar contrasts in speech. Musical training contributes to the establishment of cognitive enhancement, such that there are some enduring cross-domain transfer benefits of musical training on the discrimination of subtle speech contrasts even after musical training and practice is discontinued.

More importantly, our finding that former musicians differed from active musicians qualifies the extent of the positive music-to-language transfer effects. Drawing on previous studies showing a clear behavioural advantage held by adult musicians over non-musicians in a pitch contour violation task similar to that used in the present study^31,34^, it appears that former musicians retain some musician advantage, but that such advantage may fade over time after musical training and practice is discontinued. Parallel results were seen in the data from the musical abilities tests. There were significant group differences in MET melody scores (Table 1), where active musicians had better musical abilities than former musicians, and former musicians in turn had better musical abilities than non-musicians. The difference in pitch discrimination and musical abilities between active musicians and former musicians cannot be attributed to the length of musical training, which was similar for the two groups as seen in the pairwise comparison (Table 1). Therefore, our data suggests that music attrition in pitch perception manifests in both the music and language domains among former musicians. One possible explanation is that subtle acoustic cues may no longer be behaviourally relevant in former musicians’ everyday auditory environment, such that positive music-to-language transfer benefits may diminish over time. This interpretation is corroborated by exploratory Pearson correlations conducted to assess the relationship between the number of years since discontinuing musical practice and other factors. As reported in the Supplementary Results, in the sample of musicians both active and former, the number of years since discontinuing musical practice was significantly negatively correlated with pitch discrimination accuracy for the weakly incongruous prosodic condition in the native language English and non-native language French (see Supplementary Figs. 1 and 2), as well as with MET melody and rhythm subtest performance. The longer the period since discontinuing musical training and practice, the poorer one is at discriminating subtle violations in speech and music, indicating that there might be a gradual attenuation in finer acoustic discrimination abilities among former musicians. Future research can be conducted with older adults as participants, including former musicians who have discontinued musical training and practice for a longer period, to examine the issue further.

We also found a significant main effect for language and a significant interaction effect between language and prosodic congruity. Apart from a native language context with multiple possible sources of information that might assist in pitch processing, we implemented a non-native language context without additional information for participants to rely on. The interaction effect revealed that in both the congruous and weakly incongruous conditions, participants were more accurate in prosodic pitch discrimination in their native language than in a non-native language, consistent with previous findings by Marques et al.^34^ and Deguchi et al.^35^. There are two possible explanations for this native language advantage, which Deguchi et al.^35^ investigated by introducing jabberwocky sentences that preserved the intonational contours of the native language but consisted of meaningless legal pseudowords. They found that participants were better at detecting pitch changes in their native language than in jabberwocky, but were also better at detecting pitch changes in jabberwocky than in the non-native language. This suggests that participants were familiar with typical intonational contours in their native language, and were consequently better able to detect pitch changes in the native language and jabberwocky speech stimuli but not in the non-native language speech stimuli. At the same time, participants could understand the meaning of their native language, and for that reason were better able to anticipate when the final word carrying the pitch variation would occur in the native language speech stimuli but not in the jabberwocky and non-native language speech stimuli. The native language advantage observed in our study can thus be explained by the fact that participants were making use of both prosodic and semantic information to complete the pitch incongruity detection task. Nonetheless, regardless of the language used for the speech stimuli, group differences were seen in the weakly incongruous condition. Active and former musicians were more accurate than non-musicians in detecting prosodic pitch violation no matter whether they had prior knowledge of the language tested. In the non-native language context which better isolated the prosodic pitch dimension without influence from other types of linguistic processing, participants would not have been able to exploit additional top-down processing frameworks and would have been relying solely on bottom-up pitch perceptual sensitivity to acoustic cues. In the native language context, group differences were also seen despite the fact that all participants were able to draw on additional linguistic resources. The present study hence extends the OPERA hypothesis^4^ by underscoring that—presumably due to their prior musical training with melodic pitch patterns—former musicians retain enhanced underlying pitch processing abilities, which generalise to the perception of prosodic pitch contours in speech for both native and non-native languages.

A caveat to keep in mind, though, is that although there is a large body of evidence in which musicians—be it active and former—outperform non-musicians in various pitch perception tasks, it may not be straightforward to conclude that the so-called musician advantage is a result of musical training. It is plausible that the results reported in this present study may be driven by a third, unexplored factor, such as general intelligence, education background, or socioeconomic status. On top of that, as with most of the previously reported literature, this present study adopted a cross-sectional design comparing different population groups at a specific point in time. In recent years, some researchers have propounded the idea that inherent musical abilities, rather than musical training, might be linked to enhanced speech perception^30,85,86^. Ergo, the music-to-language transfer effects that we speak of may be a consequence of pre-existing differences and self-selection, as opposed to a consequence of musical training per se. In other words, individuals pre-disposed with superior auditory or pitch processing abilities to begin with may be more inclined to pick up and continue musical training, such that the differences observed between active, former, and non-musicians later in life may not be a direct outcome of musical training in and of itself.

However, as highlighted in the introduction, there is some compelling evidence in the existing literature that musical training has a causal influence on brain development and pitch perception. Participants initially matched in musical aptitude, general intelligence, and socioeconomic status have been shown to demonstrate group differences in neurological and behavioural post-tests related to pitch perception depending on the training they are randomly assigned to^33,45–47^. Of particular relevance to our study, Moreno et al.^33^ conducted a longitudinal experimental study with 8-year-old children without any prior musical training. Pre-tests confirmed that the children were initially matched in pitch perception performance, general cognitive abilities, as well as socioeconomic status. These children were then randomly assigned to receive 6 months of either musical training or painting training. The researchers recorded both electrophysiological and behavioural measures for a pitch violation discrimination task similar to that used in this present study. They found that children who received musical training, but not those who received painting training, showed improved prosodic pitch discrimination abilities in speech. Along the same lines, Nan et al.^45^ randomly assigned 4- to 5-year-old children with tone language experience to receive 6 months of piano training, reading training, or no training. Although the children were initially matched in general cognitive abilities and socioeconomic status, and although all groups showed improvements in general cognitive abilities, only those who received piano training showed enhanced cortical responses to pitch changes in music and speech which were correlated with behavioural performance. These findings suggest that musical training can indeed cause experience-dependent transfer effects that cannot be attributed to external factors or pre-existing differences, while our study further suggests that some transfer effects may be retained even after musical training and practice is discontinued. Having said that, future research can strengthen our finding by performing an intervention study with longitudinal randomised controlled trials to track and compare the effects of long-term, short-term, and no musical training among individuals who are otherwise matched on other variables.

In conclusion, our study shows that musical training confers positive cross-domain benefits in speech perception, adding to the body of literature on music-to-language transfer and suggesting that there is a common pitch processing mechanism underlying pitch perception in the two domains. More importantly, our results further show that these benefits may be retained to some extent over time, such that former musicians show some behavioural advantage over non-musicians even after the discontinuation of musical training and practice. Situated within the OPERA hypothesis^4^, it appears that musical training alters the shared neural networks for music and speech in a long-lasting manner, such that the musician advantage applies not only to active musicians but to former musicians as well. Moreover, this advantage in prosodic pitch perception is seen with both native and non-native languages. Possible future directions for research include using neurological and behavioural measures to compare active musicians, former musicians, and non-musicians’ pitch perception abilities in the language domain in other areas such as the perception of lexical tones, stress, and emotional prosody. Our findings have real-life implications for boosting first language acquisition and foreign language learning, as well as protecting against age-related cognitive and auditory decline in the ageing population. It appears that musical training and practice can serve as an effective enrichment activity and intervention method to improve speech perception, and that individuals can reap some long-lasting cognitive benefits throughout their lifespan even after musical training and practice is discontinued.

## Supplementary Information

Supplementary Information 1.

Supplementary Information 2.

## Data Availability

The dataset generated during and/or analysed during the current study is included in the Supplementary Information file.

## References

[1] Schellenberg EG, Weiss WM, Deutsch D (2013). Music and cognitive abilities. The Psychology of Music.

[2] Parbery-Clark A, Anderson S, Hittner E, Kraus N (2012). Musical experience offsets age-related delays in neural timing. Neurobiol. Aging.

[3] Parbery-Clark A, Skoe E, Lam C, Kraus N (2009). Musician enhancement for speech-in-noise. Ear. Hear..

[4] Patel AD (2011). Why would musical training benefit the neural encoding of speech? The OPERA hypothesis. Front. Psychol..

[5] Alexander, J. A., Wong, P. C. M. & Bradlow, A. R. Lexical tone perception in musicians and non-musicians. In *Proceedings of Proc. Annual Conference of the International Speech Communication Association Interspeech* (2005).

[6] Burnham D, Brooker R, Reid A (2015). The effects of absolute pitch ability and musical training on lexical tone perception. Psychol. Music..

[7] Choi W (2020). The selectivity of musical advantage: Musicians exhibit perceptual advantage for some but not all Cantonese tones. Music Percept..

[8] Delogu F, Lampis G, Belardinelli MO (2010). From melody to lexical tone: Musical ability enhances specific aspects of foreign language perception. Eur. J. Cogn. Psychol..

[9] Gottfried TL, Riester D (2000). Relation of pitch glide perception and Mandarin tone identification. J. Acoust. Soc. Am..

[10] Gottfried TL, Staby AM, Ziemer CJ (2001). Musical experience and Mandarin tone discrimination and imitation. J. Acoust. Soc. Am..

[11] Han Y, Goudbeek M, Mos M, Swerts M (2019). Mandarin tone identification by tone-naïve musicians and non-musicians in auditory-visual and auditory-only conditions. Front. Commun..

[12] Hung, T.-H. & Lee, C.-Y. Processing linguistic and musical pitch by English-speaking musicians and non-musicians. In *20th North American Conference on Chinese Linguistics *(2008).

[13] Lee C-Y, Hung T-H (2008). Identification of Mandarin tones by English-speaking musicians and nonmusicians. J. Acoust. Soc. Am..

[14] Marie CL, Delogu F, Lampis G, Belardinelli MO, Besson M (2011). Influence of musical expertise on segmental and tonal processing in Mandarin Chinese. J. Cogn. Neurosci..

[15] Wayland RP, Herrera E, Kaan E (2010). Effects of musical experience and training on pitch contour perception. J. Phon..

[16] Bidelman GM, Gandour JT, Krishnan A (2011). Cross-domain effects of music and language experience on the representation of pitch in the human auditory brainstem. J. Cogn. Neurosci..

[17] Wong PCM, Skoe E, Russo NM, Dees T, Kraus N (2007). Musical experience shapes human brainstem encoding of linguistic pitch patterns. Nat. Neurosci..

[18] Mok PPK, Zuo D (2012). The separation between music and speech: Evidence from the perception of Cantonese tones. J. Acoust. Soc. Am..

[19] Ong JH, Wong PCM, Liu F (2020). Musicians show enhanced perception, but not production, of native lexical tones. J. Acoust. Soc. Am..

[20] Toh XR, Lau F, Wong FCK (2022). Individual differences in nonnative lexical tone perception: Effects of tone language repertoire and musical experience. Front. Psychol..

[21] Kolinsky R, Cuvelier H, Goetry V, Peretz I, Morais J (2009). Music training facilitates lexical stress processing. Music Percept..

[22] Choi W (2022). Towards a native OPERA hypothesis: Musicianship and English stress perception. Lang. Speech.

[23] Choi W (2022). What is "music" in music-to-language transfer? Musical ability but not musicianship supports Cantonese listeners' English stress perception. J. Speech Lang. Hear. Res..

[24] Lee Y-S, Vakoch DA, Lee HW (1996). Tone perception in Cantonese and Mandarin: A cross-linguistic comparison. J. Psycholinguist. Res..

[25] Morett LM (2020). The influence of tonal and atonal bilingualism on children’s lexical and non-lexical tone perception. Lang. Speech.

[26] Qin Z, Mok PKP (2013). Discrimination of Cantonese tones by speakers of tone and non-tone languages. Kans. Work. Pap. Linguist..

[27] Schaefer V, Darcy I (2014). Lexical function of pitch in the first language shapes cross-linguistic perception of Thai tones. Lab. Phonol..

[28] Schaefer V, Darcy I (2020). Applying a newly learned second language dimension to the unknown: The influence of second language Mandarin tones on the naïve perception of Thai tones. Psychol. Lang. Commun..

[29] Wayland RP, Guion SG (2004). Training English and Chinese listeners to perceive Thai tones: A preliminary report. Lang. Learn..

[30] Wayland RP, Li B (2008). Effects of two training procedures in cross-language perception of tones. J. Phon..

[31] Schön D, Magne C, Besson M (2004). The music of speech: Music training facilitates pitch processing in both music and language. Psychophysiology.

[32] Magne C, Schön D, Besson M (2006). Musician children detect pitch violations in both music and language better than nonmusician children: Behavioral and electrophysiological approaches. J. Cogn. Neurosci..

[33] Moreno S (2009). Musical training influences linguistic abilities in 8-year-old children: More evidence for brain plasticity. Cereb. Cortex.

[34] Marques C, Moreno S, Castro SL, Besson M (2007). Musicians detect pitch violation in a foreign language better than nonmusicians: Behavioral and electrophysiological evidence. J. Cogn. Neurosci..

[35] Deguchi C (2012). Sentence pitch change detection in the native and unfamiliar language in musicians and non-musicians: Behavioral, electrophysiological and psychoacoustic study. Brain Res..

[36] Thompson WF, Schellenberg EG, Husain G (2003). Perceiving prosody in speech: Effects of music lessons. Ann. N. Y. Acad. Sci..

[37] Thompson WF, Schellenberg EG, Husain G (2004). Decoding speech prosody: Do music lessons help?. Emotion.

[38] Farmer E, Jicol C, Petrini K (2020). Musicianship enhances perception but not feeling of emotion from others’ social interaction through speech prosody. Music Percept..

[39] Lima CF, Castro SL (2011). Speaking to the trained ear: Musical expertise enhances the recognition of emotions in speech prosody. Emotion.

[40] Pantev C (1998). Increased auditory cortical representation in musicians. Nature.

[41] Pantev C, Engelien A, Candia V, Elbert T (2001). Representational cortex in musicians: Plastic alterations in response to musical practice. Ann. N. Y. Acad. Sci..

[42] Schlaug G (2001). The brain of musicians: A model for functional and structural adaptation. Ann. N. Y. Acad. Sci..

[43] Bermudez P, Zatorre RJ (2005). Differences in gray matter between musicians and nonmusicians. Ann. N. Y. Acad. Sci..

[44] Gaser C, Schlaug G (2003). Brain structures differ between musicians and non-musicians. J. Neurosci..

[45] Nan Y (2018). Piano training enhances the neural processing of pitch and improves speech perception in Mandarin-speaking children. Proc. Natl. Acad. Sci. USA.

[46] Hyde KL (2009). The effects of musical training on structural brain development. Ann. N. Y. Acad. Sci..

[47] Hyde KL (2009). Musical training shapes structural brain development. J. Neurosci..

[48] Kraus N, Skoe E, Parbery-Clark A, Ashley R (2009). Experience-induced malleability in neural encoding of pitch, timbre, and timing. Ann. N. Y. Acad. Sci..

[49] Wan CY, Schlaug G (2010). Music making as a tool for promoting brain plasticity across the lifespan. Neuroscientist.

[50] Neves L, Correia AI, Castro SL, Martins D, Lima CF (2022). Does music training enhance auditory and linguistic processing? A systematic review and meta-analysis of behavioral and brain evidence. Neurosci. Biobehav. Rev..

[51] Zendel BR, Alain C (2012). Musicians experience less age-related decline in central auditory processing. Psychol. Aging.

[52] Zendel BR, Alain C (2013). The influence of lifelong musicianship on neurophysiological measures of concurrent sound segregation. J. Cogn. Neurosci..

[53] Alain C, Zendel BR, Hutka S, Bidelman GM (2014). Turning down the noise: The benefit of musical training on the aging auditory brain. Hear. Res..

[54] Zhang JD, Susino M, McPherson GE, Schubert E (2020). The definition of a musician in music psychology: A literature review and the six-year rule. Psychol. Music.

[55] Costa-Giomi E, MacDonald R, Kreutz G, Mitchell L (2012). Music instruction and children’s intellectual development: The educational context of music participation. Music, Health, and Wellbeing.

[56] Costa-Giomi E (2015). The long-term effects of childhood music instruction on intelligence and general cognitive abilities. Update Appl. Res. Music Educ..

[57] Costa-Giomi E, Ryan C (2007). The benefits of music insturction: What remains years later. Symp. Res. Music Behav..

[58] Costa-Giomi E (1999). The effects of three years of piano instruction on children’s cognitive development. J. Res. Music Educ..

[59] Schellenberg EG (2006). Long-term positive associations between music lessons and IQ. J. Educ. Psychol..

[60] Strong JV, Midden A (2020). Cognitive differences between older adult instrumental musicians: Benefits of continuing to play. Psychol. Music.

[61] Skoe E, Kraus N (2012). A little goes a long way: How the adult brain is shaped by musical training in childhood. J. Neurosci..

[62] White-Schwoch T, Carr KW, Anderson S, Strait DL, Kraus N (2013). Older adults benefit from music training early in life: Biological evidence for long-term training-driven plasticity. J. Neurosci..

[63] Wennerstrom A (2001). The Music of Everyday Speech: Prosody and Discourse Analysis.

[64] Patel AD, Wong M, Foxton J, Lochy A, Peretz I (2008). Speech intonation perception deficits in musical tone deafness (congenital amusia). Music Percept..

[65] Hutchins S, Gosselin N, Peretz I (2010). Identification of changes along a continuum of speech intonation is impaired in congenital amusia. Front. Psychol..

[66] Jiang C, Hamm JP, Lim VK, Kirk IJ, Yang Y (2010). Processing melodic contour and speech intonation in congenital amusics with Mandarin Chinese. Neuropsychologia.

[67] Liu F, Patel AD, Fourcin A, Stewart L (2010). Intonation processing in congenital amusia: Discrimination, identification and imitation. Brain.

[68] Monrad-Krohn GH (1947). The prosodic quality of speech and its disorders. Acta Psychiatr. Scand..

[69] Zhang J, McBride-Chang C (2010). Auditory sensitivity, speech perception, and reading development and impairment. Educ. Psychol. Rev..

[70] Holliman AJ, Williams GJ, Mundy IR, Wood C, Hart L, Waldron S (2014). Beginning to disentangle the prosody-literacy relationship: A multi-component measure of prosodic sensitivity. Read. Writ..

[71] Groen MA, Veenendaal NJ, Verhoeven L (2019). The role of prosody in reading comprehension: Evidence from poor comprehenders. J. Res. Read..

[72] Cutler A, Dahan D, van Donselaar W (1997). Prosody in the comprehension of spoken language: A literature review. Lang. Speech.

[73] Hellbernd N, Sammler D (2016). Prosody conveys speaker’s intentions: Acoustic cues for speech act perception. J. Mem. Lang..

[74] Hupp JM, Jungers MK, Hinerman CM, Porter BL (2021). Cup! Cup? Cup: Comprehension of intentional prosody in adults and children. Cogn. Dev..

[75] Campfield DE, Murphy VA (2017). The influence of prosodic input in the second language classroom: Does it stimulate child acquisition of word order and function words?. Lang. Learn. J..

[76] Saksida A, Fló A, Guedes B, Nespor M, Peña Garay M (2021). Prosody facilitates learning the word order in a new language. Cognition.

[77] Saito Y, Saito K (2017). Differential effects of instruction on the development of second language comprehensibility, word stress, rhythm, and intonation: The case of inexperienced Japanese EFL learners. Lang. Teach. Res..

[78] Yenkimaleki M (2021). Prosody training benefits in perception vs production skills in simultaneous interpreting: An experimental study. Dutch J. Appl. Linguist..

[79] Wallentin M, Nielsen AH, Friis-Olivarius M, Vuust C, Vuust P (2010). The Musical Ear Test, a new reliable test for measuring musical competence. Learn. Indiv. Differ..

[80] Rothauser EH (1969). IEEE recommended practice for speech quality measures. IEEE Trans. Audio Electroacoust..

[81] Smith CL (2002). Prosodic finality and sentence type in French. Lang. Speech.

[82] Audacity: Free Audio Editor and Recorder v. 2.3.2 (2018).

[83] Boersma P (2001). Praat, a system for doing phonetics by computer. Glot Int..

[84] Krizman J, Marian V, Shook A, Skoe E, Kraus N (2012). Subcortical encoding of sound is enhanced in bilinguals and relates to executive function advantages. Proc. Natl. Acad. Sci. USA.

[85] Mankel K, Bidelman GM (2018). Inherent auditory skills rather than formal music training shape the neural encoding of speech. Proc. Natl. Acad. Sci. USA.

[86] Swaminathan S, Schellenberg EG (2020). Musical ability, music training, and language ability in childhood. J. Exp. Psychol. Learn. Mem. Cogn..

