# Supplementary material for: Enduring musician advantage among former musicians in prosodic pitch perception
Source: Sci Rep. 2023 Feb 14;13:2657. doi: 10.1038/s41598-023-29733-3 (PMC9929097; doi:10.1038/s41598-023-29733-3)

**Supplementary Table 1.** List of English experimental speech stimuli.

| **S/N** | **Sentence** |
| --- | --- |
| 1 | Glue the sheet to the dark blue background. |
| 2 | A rod is used to catch pink salmon. |
| 3 | A pot of tea helps to pass the evening. |
| 4 | The stray cat gave birth to kittens. |
| 5 | The young girl gave no clear response. |
| 6 | The play seems dull and quite stupid. |
| 7 | Ten pins were set in order. |
| 8 | The cup cracked and spilled its contents. |
| 9 | There was a sound of dry leaves outside. |
| 10 | Live wires should be kept covered. |
| 11 | They took the axe and the saw to the forest. |
| 12 | Some ads serve to cheat buyers. |
| 13 | A waxed floor makes us lose balance. |
| 14 | Nine men were hired to dig the ruins. |
| 15 | Watch the log float in the wide river. |
| 16 | Write fast if you want to finish early. |
| 17 | Let's all join as we sing the last chorus. |
| 18 | The fight will end in just six minutes. |
| 19 | Our plans right now are hazy. |
| 20 | Grace makes up for lack of beauty. |
| 21 | The boy owed his friend ten dollars. |
| 22 | He put his last cartridge into the gun and fired. |
| 23 | I have to do laundry while it’s still sunny. |
| 24 | She takes the dog for a run in the garden. |
| 25 | Parents must also become independent of their children. |
| 26 | Meeting my old friend was very pleasant. |
| 27 | This day was just a waste of time and money. |
| 28 | I cannot put up with his idleness any longer. |
| 29 | I really like strawberries, oranges and lemons. |
| 30 | I eat bread with cheese for breakfast. |
| 31 | Someone said that she will leave for Spain in winter. |
| 32 | It is well said that time is anger’s medicine. |
| 33 | He sent the boy on a short errand. |
| 34 | When you hear the bell, come quickly. |
| 35 | He had four cups of coffee. |
| 36 | The baby's feet were cute and tiny. |
| 37 | We ate our dinner in a noisy cafe. |
| 38 | The cow died from a sudden illness. |
| 39 | My father loves to listen to the radio. |
| 40 | The doctor prescribed me cough syrup. |

**Supplementary Table 2.** List of French experimental speech stimuli.

| **S/N** | **Sentence** | **Translation** |
| --- | --- | --- |
| 1 | Ma maman m’a demandé hier d’inviter mon parrain. | Yesterday, my mother asked me to invite my godfather. |
| 2 | Sa longue jupe rouge est vraiment très facile à porter. | Her long, red skirt is really very easy to wear. |
| 3 | Tous les jours, la pie donnait à manger à ses petits. | The magpie gave food to its babies every day. |
| 4 | Le cou du chien est entouré d’un magnifique collier. | The dog’s neck is encircled by a beautiful collar. |
| 5 | Le journaliste dit que La Coupe du monde aura lieu le mois prochain. | The reporter said that the World Cup will take place next month. |
| 6 | Comme prévu, elle a eu le coup de foudre pour la maison. | As expected, she fell in love with the house at first sight. |
| 7 | C’est atypique pour une région montagneuse comme celle-ci. | It’s atypical for a mountainous region like this. |
| 8 | Je crois que je t’aime seulement parce que tu es futé(e). | I think I like you only because you’re clever. |
| 9 | Chaque dimanche matin, j’emmène le chien courir dans le jardin. | Every Saturday morning, I take the dog for a run in the garden. |
| 10 | Son petit ami l’a quitté et elle s'est endormie en pleurant. | She cried herself to sleep after her boyfriend broke up with her. |
| 11 | Finir le travail pour mardi sera du gâteau. | Finishing the job by Tuesday will be a piece of cake. |
| 12 | Les parents doivent aussi apprendre à se séparer de leurs enfants. | Parents must also become independent of their children. |
| 13 | Ce fut un plaisir de rencontrer un vieux copain. | Meeting my old friend was very pleasant. |
| 14 | Cette journée n’a été qu’un gâchis de temps et d’argent. | This day was just a waste of time and money. |
| 15 | Ce n'est pas le genre de musique sur lequel on peut danser. | It’s not the type of music that you can dance to. |
| 16 | Parfois je me dis qu'on ne se reparlera jamais. | Sometimes I think I will never talk to him/her again. |
| 17 | Un bon régime alimentaire donne des bébés en meilleure santé. | A good diet makes for healthier babies. |
| 18 | C’est évident qu’elle est en train de rattraper le temps perdu. | It’s clear that she is making up for lost time now. |
| 19 | Pour être franc, le plus facile serait que tu nous montres le chemin. | To be frank, the easiest thing would be for you to lead the way. |
| 20 | Je ne peux supporter son oisiveté plus longtemps. | I cannot put up with his idleness any longer. |
| 21 | J'aime beaucoup les fraises, les citrons et aussi les pastèques. | I really like strawberries, lemons, and also watermelons. |
| 22 | Personne dans l’équipe n’a trouvé qu'il avait fait du bon travail. | Nobody in the team thought he did a good job. |
| 23 | Ça fait un certain temps que je n’ai pas rangé(e) mon placard. | It has been quite some time that I haven’t tidied my cupboard. |
| 24 | Comme mon frère, j'adore faire la grasse matinée le dimanche. | Like my brother, I love sleeping in on Sunday mornings. |
| 25 | Samedi dernier, on a eu un pépin avec la voiture. | Last Saturday, we had a bit of bad luck with the car. |
| 26 | Pour le petit déjeuner, je mange du pain avec du fromage. | I eat bread with cheese for breakfast. |
| 27 | C’est curieux, les copies de cette classe sont toujours très mauvaises. | It’s strange, this class’ papers are always very bad. |
| 28 | Les deux jeunes garçons font du vélo près du long canal. | The two boys are cycling next to the long canal. |
| 29 | Quelqu’un a dit qu’elle partira pour l’Espagne cet hiver. | Someone said that she will leave for Spain in winter. |
| 30 | L'éducation représente plus que d'aller à l'école. | Education means something more than going to school. |
| 31 | Nous nous sommes vraiment rencontrés au magasin par hazard. | It was really just an accident that we met at the store. |
| 32 | Elle serait venue volontiers mais elle était en vacances. | She would have loved to come had she not been away on vacation. |
| 33 | Je le lui ai dit, mais il ne voulait rien entendre. | I told him but he wouldn’t listen. |
| 34 | Pour se rafraîchir les idées, il s’allonge dans un pré tranquille. | He is lying down in a tranquil meadow to clear his head. |
| 35 | Elle est si jolie que tout le monde se retourne sur son passage. | She is so pretty that she attracts attention wherever she goes. |
| 36 | J’ai du mal à croire qu’il n'a jamais raconté de mensonge. | I find it hard to believe that he has never told a lie. |
| 37 | Les éléphants sont forts, courageux et défendent leur compagne. | Elephants are strong and courageous, and they defend their mates. |
| 38 | Ma montre et mon pull préféré ont disparu depuis une semaine. | My watch and my favourite sweater have been missing for a week. |
| 39 | Si vous ne saviez pas, notre bureau est du côté nord de l'immeuble. | In case you didn’t know, our office is on the northern side of the building. |
| 40 | On dit que le temps est le remède contre la colère. | It is well said that time is anger’s medicine. |

**Supplementary Results.**

Pearson correlations were conducted to assess the relationship between the number of years since discontinuing musical practice and other factors. In the sample of musicians both active and former (*n* = 49), the number of years since discontinuing musical practice was significantly negatively correlated with pitch discrimination accuracy for the weakly incongruous prosodic condition in the native language English (*r* = -0.394, *p* = 0.005) and non-native language French (*r* = -0.359, *p* = 0.011), as well as with MET melody subtest performance (*r* = -0.325, *p* = 0.023) and MET rhythm subtest performance (*r* = -0.334, *p* = 0.019).

**Supplementary Figure 1.** Correlation (Pearson’s r) between number of years since discontinuing musical practice and pitch discrimination accuracy for the weakly incongruous condition in English. ***p* < 0.01.


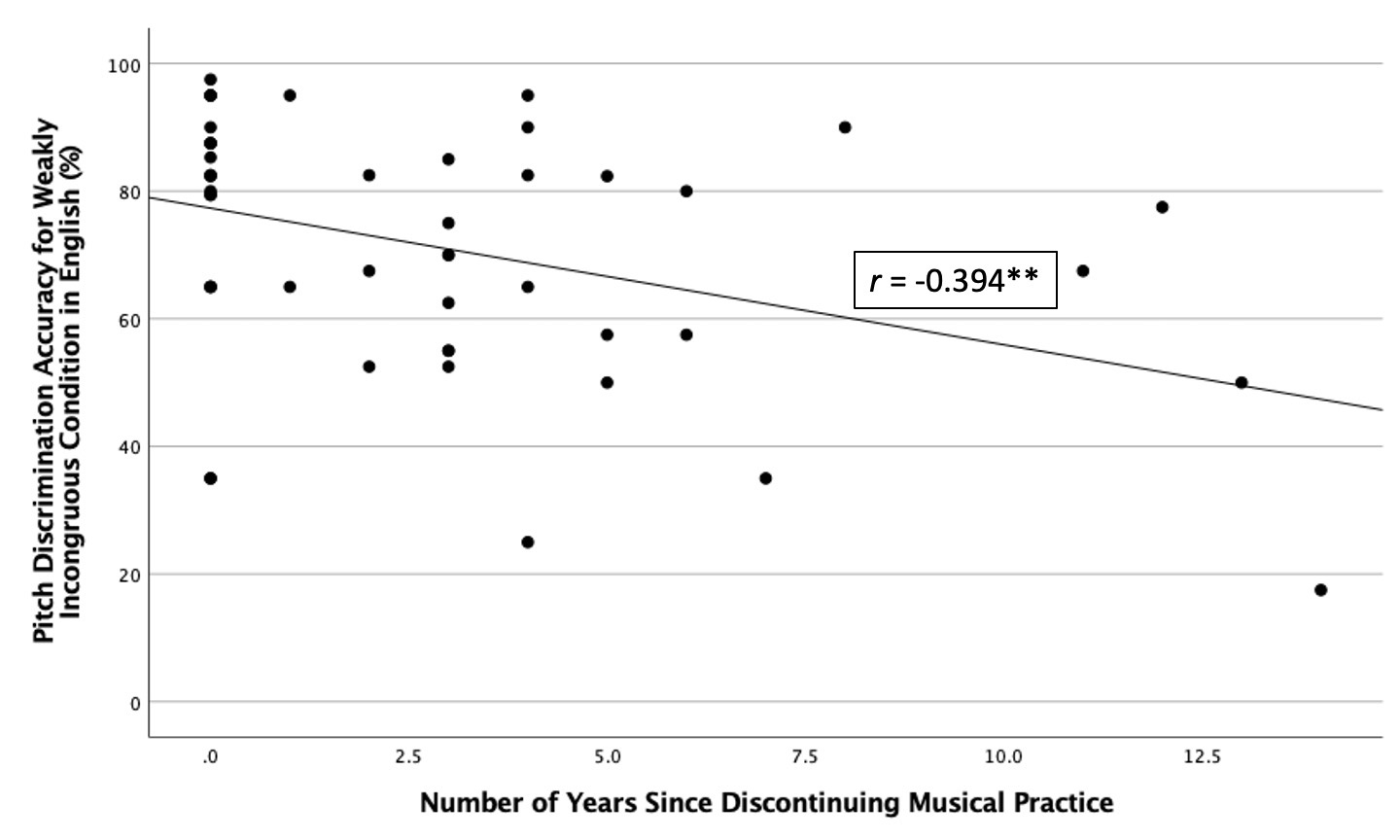


**Supplementary Figure 2.** Correlation (Pearson’s r) between number of years since discontinuing musical practice and pitch discrimination accuracy for the weakly incongruous condition in French. **p* < 0.05.


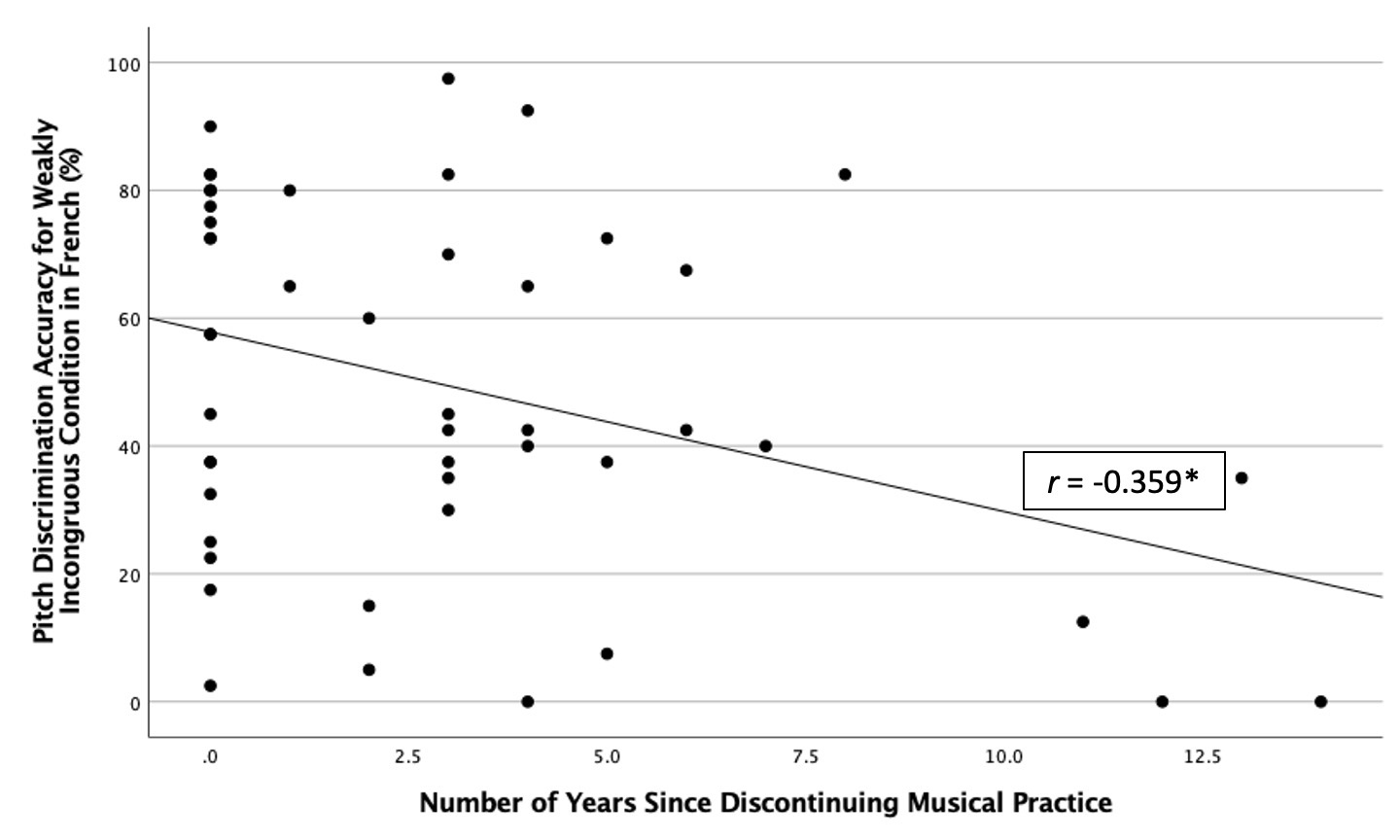

Supplement: Supplementary file 1 — Supplementary Information 1. [file 41598_2023_29733_MOESM1_ESM.docx]
